# Structural Aspects of *Mycobacterium tuberculosis* DNA Gyrase Targeted by Novel Bacterial Topoisomerase Inhibitors

**DOI:** 10.1021/acsmedchemlett.4c00447

**Published:** 2024-11-22

**Authors:** Maja Kokot, Martina Hrast Rambaher, Lipeng Feng, Lesley A Mitchenall, David M Lawson, Anthony Maxwell, Tanya Parish, Nikola Minovski, Marko Anderluh

**Affiliations:** † Theory Department, Laboratory for Cheminformatics, National Institute of Chemistry, Hajdrihova 19, 1001 Ljubljana, Slovenia; ‡ Department of Pharmaceutical Chemistry, Faculty of Pharmacy, University of Ljubljana, Aškerčeva cesta 7, 1000 Ljubljana, Slovenia; § Department of Biological Chemistry, John Innes Centre, Norwich Research Park, Norwich NR4 7UH, U.K.; ∥ Department of Molecular Microbiology, John Innes Centre, Norwich Research Park, Norwich NR4 7UH, U.K.; ⊥ Department of Biochemistry & Metabolism, John Innes Centre, Norwich Research Park, Norwich NR4 7UH, U.K.; # School of Medicine, University of Washington, Seattle, Washington 98195, United States; ○ Center for Global Infectious Disease Research, Seattle Children’s Research Institute, Seattle, Washington 98105, United States

**Keywords:** NBTIs, DNA gyrase, *M. tuberculosis*, crystal structure, bifurcated halogen bonds

## Abstract

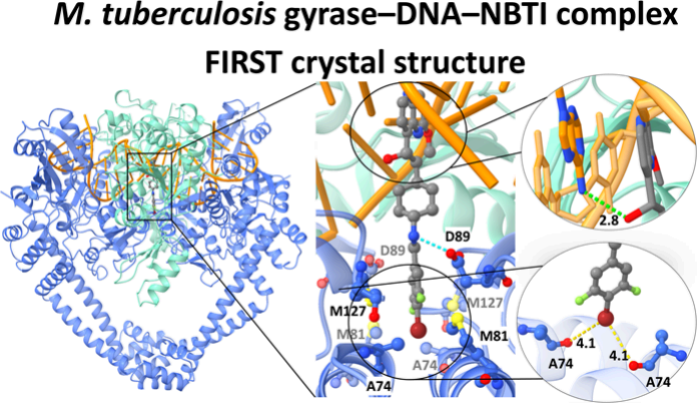

In this Letter, we present a small series of novel bacterial topoisomerase inhibitors (NTBIs) that exhibit both potent inhibition of *Mycobacterium tuberculosis* DNA gyrase and potent antimycobacterial activity. The disclosed crystal structure of *M. tuberculosis* DNA gyrase in complex with DNA and compound **5** from this NBTI series reveals the binding mode of an NBTI in the GyrA binding pocket and confirms the presence and importance of halogen bonding for the excellent on-target potency. In addition, we have shown that compound **5** is a promising *M. tuberculosis* DNA gyrase inhibitor, with an IC_50_ for *M. tuberculosis* gyrase of 0.096 μM, and it has potent activity against *M. tuberculosis*, with an IC_50_ of 0.165 μM.

Tuberculosis (TB), caused by *Mycobacterium tuberculosis*, is one of the leading causes of death worldwide. Until the coronavirus pandemic (COVID-19), TB was the leading cause of death from a single infectious agent, ahead of HIV/AIDS. The number of newly diagnosed cases of TB in 2022 was 7.5 million, and approximately 1.3 million people died from the disease.^1^ The combination therapy for TB with different antituberculosis agents has been practiced for many decades.^2^ However, the emergence of multidrug-resistant (MDR), extensively drug-resistant (XDR), and the recently identified so-called “totally” drug-resistant (TDR) TB strains has led to a global epidemic problem.^3,4^ Due to this situation, there is an urgent need for new drugs with an intensive regimen consisting of a novel mode of action.^5−7^

Topoisomerases are enzymes that maintain the correct topological state of the DNA molecule during the processes of replication, transcription, and recombination. DNA gyrase, a bacterial type II topoisomerase, is a clinically validated antibacterial target. Unlike most bacterial species that have both DNA gyrase and topoisomerase IV (topoIV), *M. tuberculosis* has only the DNA gyrase enzyme, but with a simultaneous functional role of topoIV.^8^ It performs the essential function of introducing negative supercoils into the DNA as well as decatenation activity.^9^ Structurally, DNA gyrase is a heterotetrametric enzyme with two GyrA and two GyrB subunits (A_2_B_2_). For decades, DNA gyrase has been a primary target of the fluoroquinolone antibacterials,^10^ among which moxifloxacin and levofloxacin are still two of the most valuable second-line anti-TB agents.^11^ The uncontrolled and prolonged use of fluoroquinolones has led to quinolone-caused acquired resistance due to mutations usually harbored in the so-called quinolone resistance-determining region (QRDR) of both GyrA and GyrB subunits.^12^ Fluoroquinolone-resistant *M. tuberculosis* strains showed 4–32× lower susceptibility to fluoroquinolones compared to the reference wild-type *M. tuberculosis* H37Rv strain, so new antituberculosis agents are urgently needed.^13^

A new class of antibacterials discovered nearly two decades ago is the so-called novel bacterial topoisomerase inhibitors (NBTIs), which target the same bacterial enzymes as fluoroquinolones but have distinct chemical structures, binding mechanisms, and binding sites.^13^ They consist of three main parts: a heteroaromatic “left-hand-side” (LHS), which intercalates between the central DNA base pairs; a bi/monocyclic heteroaromatic “right-hand-side” (RHS) fragment that binds into a deep, hydrophobic binding pocket assembled at the interface of both GyrA subunits; and a connecting linker moiety (Figure 1).^14,15^ Recently, various NBTIs with confirmed activity against *M. tuberculosis* DNA gyrase have been published.^16−22^ Just as this Letter was being revised, Gedeon et al. published the first-ever structural information on the binding mode and interactions of a NBTI with the DNA gyrase enzyme of *M. tuberculosis*. Their high-resolution cryo-electron microscopy structural analysis provides detailed insights into the ternary complex formed by the *M. tuberculosis* DNA gyrase, DNA, and the reported NBTI (BDM71403). The lack of structural data prior to the work of Gedeon et al. and the work presented here has hampered the antimycobacterial discovery campaigns that would rely on a suitable, three-dimensional experimental structural data, and we hope that both sets of structural data will spur new NBTI campaigns to combat mycobacterial infections.^23^

**Figure 1:**
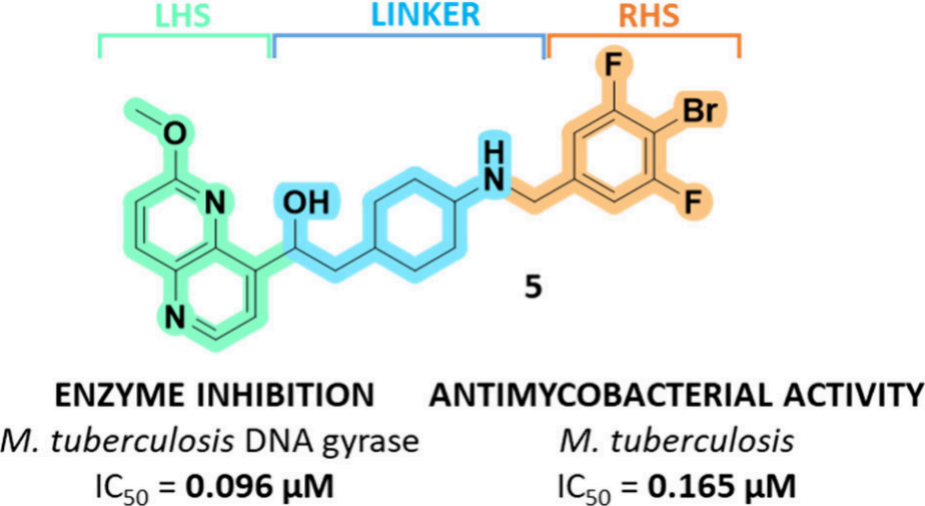
NBTI **5** showed potent enzyme inhibition and strong antituberculosis activity.

The alignment of *Staphylococcus aureus* and *M. tuberculosis* DNA gyrase amino acid sequences shows a moderate identity of 58%. Yet, the comparison of the amino acid sequences outlining the NBTI binding pocket in *S. aureus* and *M. tuberculosis* DNA gyrase revealed a high level of structural conservation of the key amino acid residues interacting with the NBTIs (Ala68, Met75, and Met121 in *S. aureus* GyrA and Ala74, Met81, and Met127 in *M. tuberculosis* GyrA). Our recently disclosed crystal structure complex of an NBTI ligand AMK-12 and *S. aureus* DNA gyrase showed that Ala68 residues from both GyrA subunits are important for the formation of symmetrical bifurcated halogen bonds, with the halogen atom at the *para* position of the phenyl RHS moiety.^24^ In the crystal structure of the *M. tuberculosis* DNA gyrase in complex with DNA and NBTI compound **5** revealed here, these alanine residues correspond to Ala74. The remaining amino acid residues (e.g., Met78, Ala78, and Met127) that comprise the NBTI’s binding pocket are mainly hydrophobic in nature and establish van der Waals interactions with the ligand’s RHS moiety.

Based on the similarity of the NBTI binding sites of *S. aureus* and *M. tuberculosis* DNA gyrase enzymes, we hypothesized that our previously published NBTIs with strong inhibitory potency on *S. aureus* DNA gyrase would also inhibit *M. tuberculosis* (Table 1). As demonstrated, all assayed compounds, in particular, **3**–**5**, exhibit potent inhibition of the enzyme in the nM concentration range as well as strong antimycobacterial activity against the wild-type *M. tuberculosis* H37Rv and fluoroquinolone-resistant LP-FQ-RM9 strain, respectively. Here, we also disclose the first crystal structure of the complex of compound **5** with *M. tuberculosis* DNA gyrase and DNA. These experimental data reinforce the concept that symmetrical bifurcated halogen-bonding interactions of NBTIs containing *p*-halogenated phenyl RHSs and backbone carbonyl oxygens of GyrA alanine residues are important for their excellent enzyme inhibitory potencies and deliver an excellent cornerstone for the structure-guided design of potent *M. tuberculosis* DNA gyrase inhibitors.

**Table 1:**
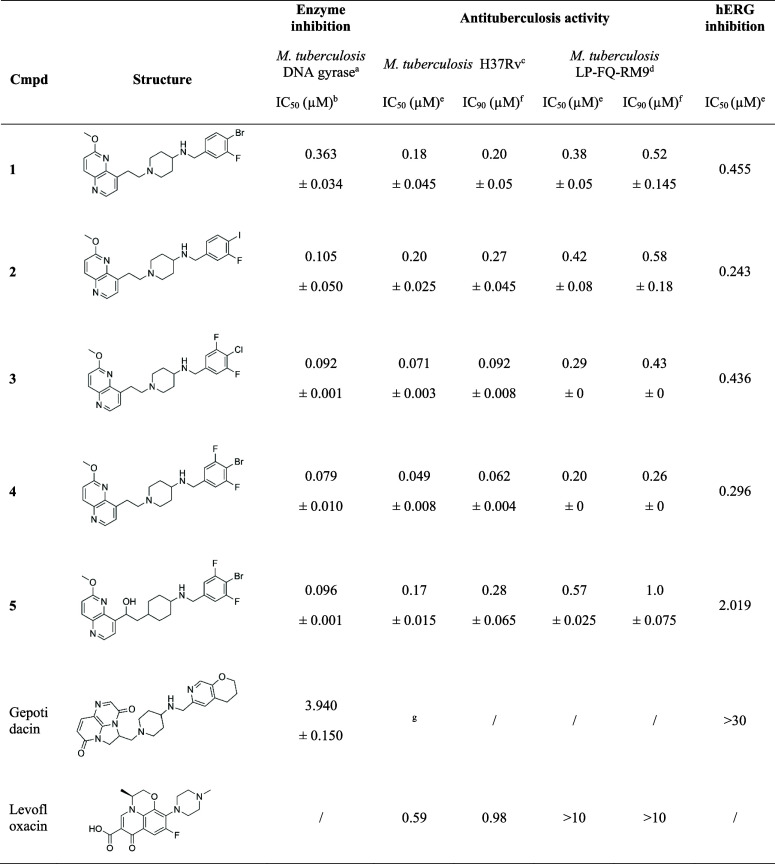
*M. tuberculosis* DNA Gyrase Inhibitory Potencies and Antimicrobial Susceptibility of the NBTIs against Reference and Fluoroquinolone-Resistant *M. tuberculosis* Strains

a *M. tuberculosis* gyrase supercoiling assays.

b IC_50_ (concentration of an inhibitor that reduces enzyme activity by 50%), mean of two independent measurements ± SD.

c *M. tuberculosis* H37Rv reference strain.

d Fluoroquinolone-resistant M. tuberculosis strain.

e IC_50_ (concentration of an inhibitor required to reach 50% inhibition of bacteria growth), mean of two independent measurements ± SD.

f IC_90_ (concentration of an inhibitor required to reach 90% inhibition of bacteria growth), mean of two independent measurements ± SD.

g MIC = 0.86 **μ**M value for Gepotidacin was calculated from the published MIC value.^26^ “/” values were not found in the literature.

## Inhibition of DNA Gyrase Supercoiling and Antituberculosis Activity

In the initial screening, 36 compounds were screened at a concentration of 100 nM using the *M. tuberculosis* DNA Gyrase Supercoiling Assay Kit (Inspiralis, Norwich, UK). Of all compounds, 28 showed a percent of inhibition at 100 nM of less than 50%, and 8 compounds showed that greater than 50% (Table S1). All eight compounds contain a *p*-halogenated RHS moiety. Similarly, Bobesh et al. previously demonstrated that NBTIs containing *p*-chlorophenyl and *p*-bromophenyl RHSs have nanomolar activities.^17^ Five compounds that exhibited greater than 80% inhibition at 100 nM were further tested to determine their IC_50_ values (Table 1). Determination of the IC_50_ values of these compounds was conducted by Inspiralis (Norwich, UK) by using the gel-based *M. tuberculosis* Gyrase Supercoiling assay (method described in experimental section and experimental data in the Supporting Information, Supplementary Table S2, and Supplementary Figures S3–S8). In comparison to the standard DNA gyrase inhibitor from the NBTI class, gepotidacin (IC_50_ = 3.940 μM), all five compounds showed 10-fold or higher enzyme inhibitory potency. Three of the five compounds showed inhibition with IC_50_ values below 100 nM (Table 1), indicating potent inhibition of *M. tuberculosis* DNA gyrase. As previously observed with the inhibition of other bacterial topoisomerases^25^ (DNA gyrase and topoIV of *S. aureus* and *E. coli*), the additional substitution of *p*-halogenated phenyl RHS with fluorine led to stronger enzyme inhibition as well as antituberculosis activity (compounds **1** to **4**). Replacement of the piperidine linker moiety with a cyclohexane linker comprising a hydroxyl group leads to a similar inhibition and antimycobacterial activity (compounds **4** and **5**). To evaluate the *in vitro* activity of the five selected compounds against *M. tuberculosis*, IC_50_ and IC_90_ values were calculated as the concentration required to reach 50% and 90% inhibition of growth (Table 1), respectively, of a reference wild-type *M. tuberculosis* H37Rv strain and a fluoroquinolone-resistant *M. tuberculosis* LP-FQ-RM9 strain (determination of antituberculosis activity is described in the Supporting Information). We have sequenced both the wild-type (H37Rv) and the GyrA mutant (LP-FQ-RM9) to ensure that they had not accumulated further mutations in GyrA. We confirmed that the H37Rv strain has no mutations and that the LP-FQ-RM9 strain has a single nucleotide polymorphism, leading to the mutation D94N, which explains the mechanistic basis for fluoroquinolone resistance. Compounds exhibited more potent activity against the reference *M. tuberculosis* strain relative to the fluoroquinolone-resistant strain. While the activity against the fluoroquinolone-resistant strain was diminished, their IC_50_ and IC_90_ values are still in the nM concentration range, in contrast to levofloxacin, where no activity was observed.

## *M. tuberculosis* DNA Gyrase–DNA–**5** Ternary Complex

**Figure 2:**
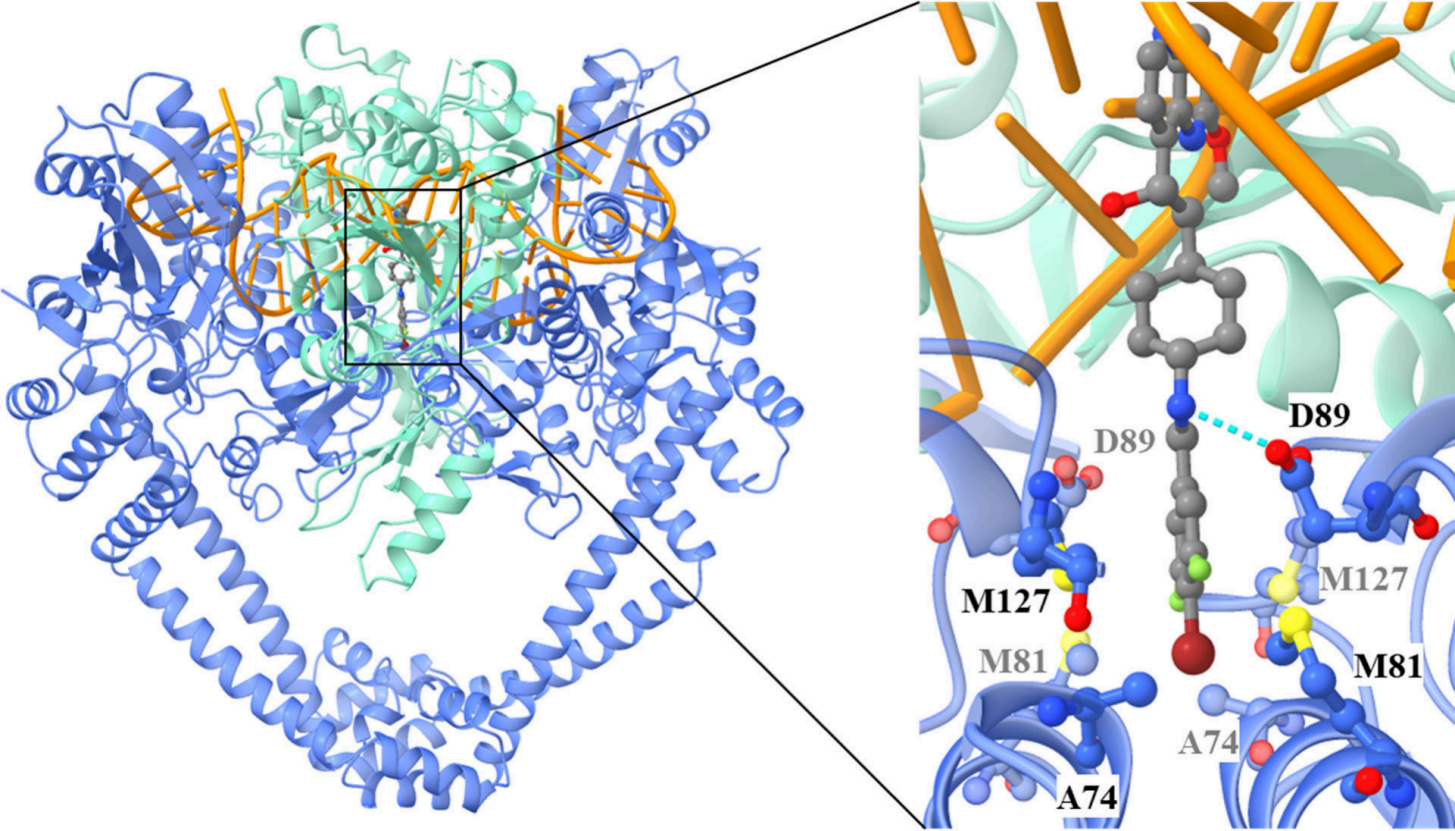
2.8 Å resolution crystal structure of compound **5** bound to *M. tuberculosis* DNA–gyrase complex (PDB ID: 9FOY). GyrA and GyrB are represented as cartoons in blue and green, **5** is represented as balls and sticks, color coded by elements, and DNA is represented as a cartoon in orange. The single hydrogen bond to the ligand in the linker part (a part of the salt bridge) is shown as a blue dashed line.

**Figure 3:**
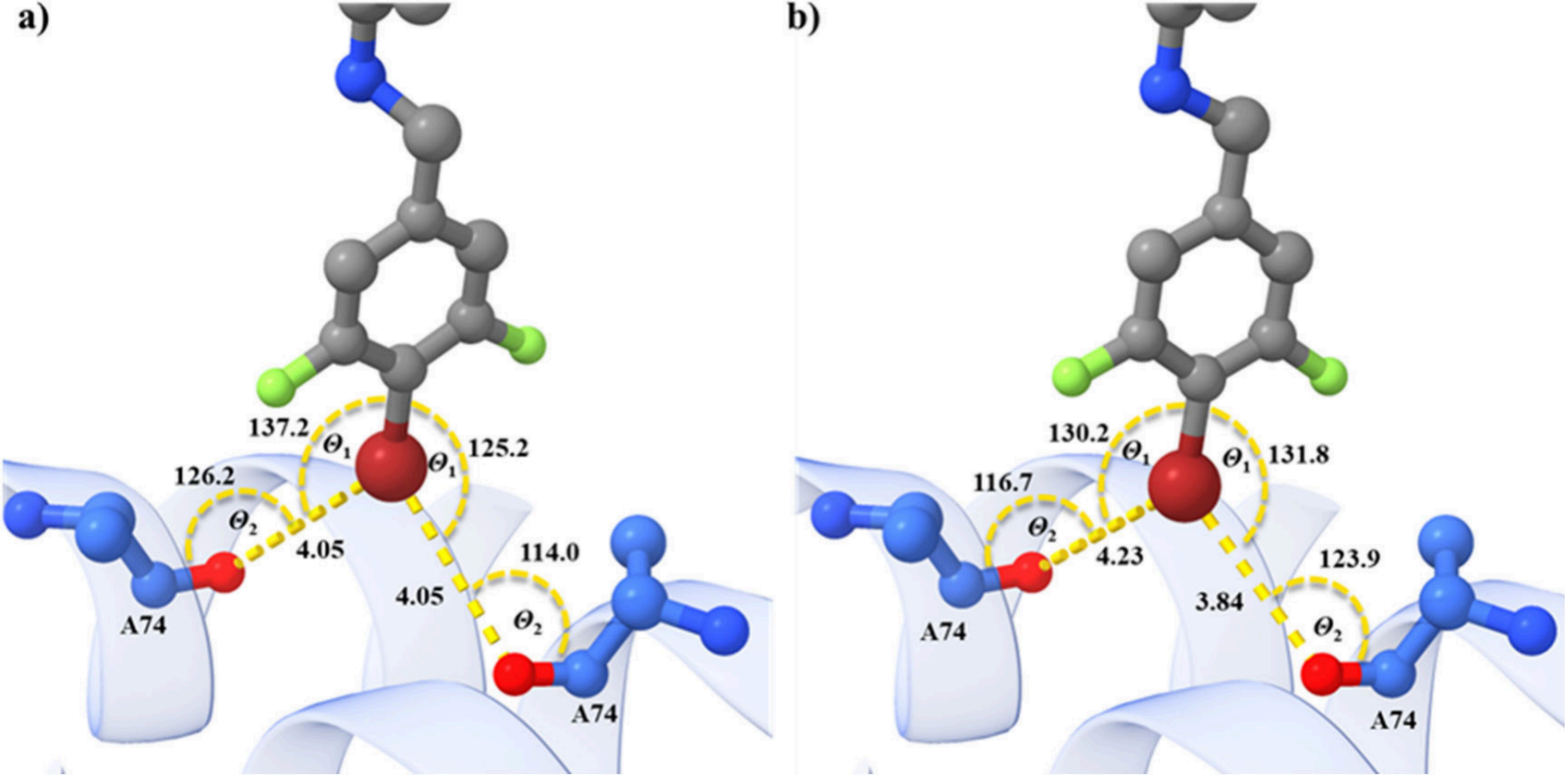
Halogen bonding in the crystal complex of compound **5** with *M. tuberculosis* DNA gyrase. Compound **5** is represented as a cartoon in blue, and DNA gyrase is represented as a cartoon in blue. Ala74 residues are in ball and stick representation, color coded by element. (a) *R* -stereoisomer: completely symmetrical bifurcated halogen bond. b) *S*-stereoisomer: less symmetrical bifurcated halogen bond.

To elucidate the binding mode of an NBTI to the DNA gyrase enzyme originating from *M. tuberculosis*, we performed crystallization studies with halogenated compound **5**. All experimental procedures and details related to protein expression, purification, crystallization, X-ray data collection, processing, and structure solution are summarized in the Supporting Information. Here, we present the first 2.8 Å resolution crystal structure of compound **5** in complex with the *M. tuberculosis* DNA gyrase core fusion and a DNA fragment. As shown in Figure 2, the LHS (methoxy-naphthyridine) moiety of compound **5** intercalates between the DNA base pairs along the 2-fold axis of the complex, positioned midway between the two DNA cleavage sites. Additionally, the 3,5-difluoro-4-bromo phenyl RHS moiety occupies a deep, hydrophobic binding pocket formed at the interface of both GyrA subunits in a similar fashion as our previously disclosed cocrystallized NBTI AMK-12 in a complex with *S. aureus* DNA gyrase.^24^ Moreover, this crystal structure once again revealed that the asymmetry of **5** (with respect to the LHS moiety) induces the asymmetry in the nicked DNA conformation, which in turn affects the configuration of the catalytic site. The compound **5** was present in the crystal structure as a racemic mixture (Figure S1), since the two enantiomers were not separated prior to the crystallization experiment, and each stereoisomer was modeled with a 50% occupancy. The 3,5-difluoro-4-bromo phenyl RHS moiety is tightly embraced by the surrounding amino acid residues in a tight pocket between the two GyrA subunits. This pocket is predominantly hydrophobic and consists of the amino acids Ala74, Ala78, Met81, and Met127, engaged primarily in van der Waals interactions with the RHS moiety. The crystal structure reveals another feature of RHS binding, namely, the halogen bonding interaction between the bromine and the backbone carbonyl oxygens of both GyrA Ala74 residues (Figure 2 and Figure 3), which provides a symmetric binding site. Both stereoisomers form bifurcated halogen bonds, with one forming fully symmetrical halogen bonds (Figure 3a) and the other forming less symmetrical halogen bonds (Figure 3b). Halogens are comparable to hydrogen by facilitating binding affinity in drug design.^27^ This is because they can be considered the hydrophobic counterparts of hydrogen bond donors, offering a clear advantage with lower desolvation penalties incurred when forming a halogen bond.^28^ This advantage is particularly pronounced in tight hydrophobic binding sites such as the one we observe at the GyrA dimer interface.

The resolved crystal structure clearly shows that in a special case where two carbonyl oxygens are involved in the halogen bond, a different symmetrical bifurcated halogen bond can be obtained. Each of the two bifurcated halogen bonds is most likely weaker than a single halogen bond due to the suboptimal geometry, but the sum of the two binding free energies is greater than that of a single bond. The existence of bifurcated halogen bonds in our previously resolved crystal structure showed that threshold values for X···O bond lengths and Θ_1_/Θ_2_ angles in halogen bonds should be redefined to detect more bifurcated halogen bonds in the crystal structures.^24^ Therefore, a new set of boundary conditions for X···O bond lengths (X···O < 4 Å) and angles (70° ≤ Θ_1_ [C–X···O] ≤ 180° and 60° ≤ Θ_2_ [X···O=C] ∼ 170°) was proposed. In the present crystal structure, one isomer forms a completely symmetrical bifurcated halogen bond where Θ_1_ and Θ_2_ angles fall within the boundary values hovering around 130° and 120°, while the X···O distance is slightly suboptimal but still within the proposed boundary values (around 4 Å). The additional substitution of the *p*-halophenyl RHS by an electron-withdrawing fluorine leads to an increase in the positive potential and the size of the σ-hole of the adjacent *p*-positioned halogen, which increases the binding free energy of the halogen bond.^29,30^ Furthermore, the size of the σ-hole is important to enable the optimal geometry for the bifurcated halogen bonding because it increases the allowable Θ_1_ angle.^24^ However, most NBTIs from other groups lack halogens in the RHS and therefore cannot form a halogen bond with the DNA gyrase, which proves that the high affinity of the RHS fragment can also be achieved without halogen bonds.

**Figure 4:**
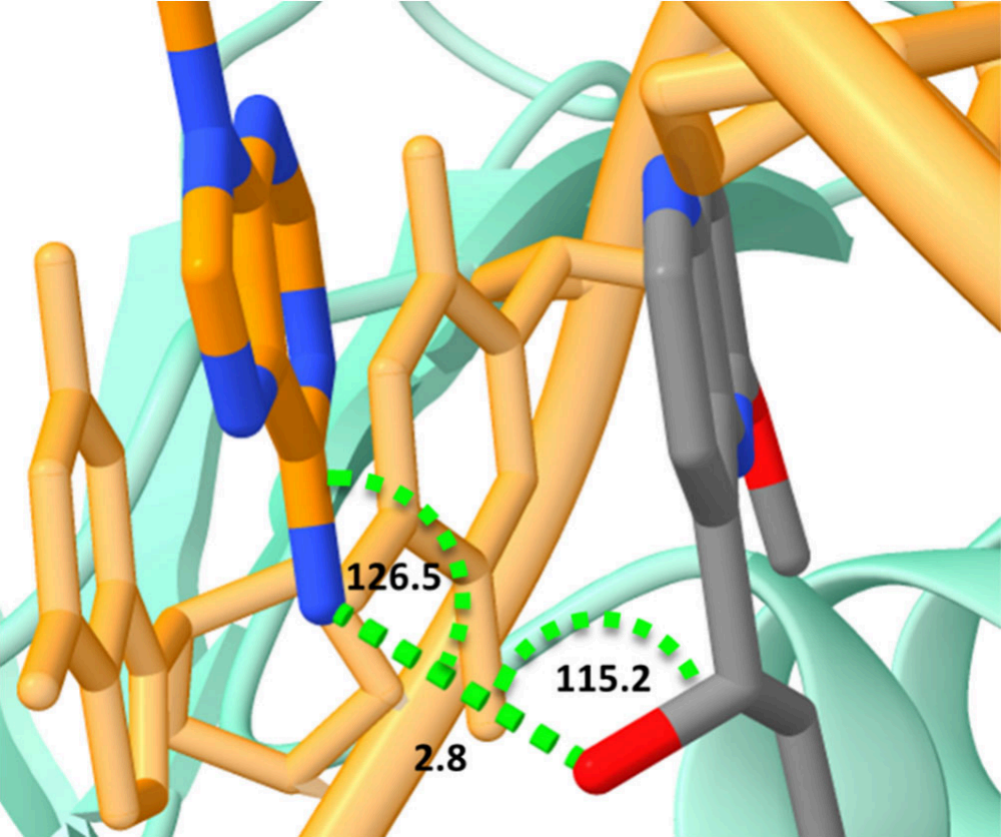
Hydrogen bond of the hydroxyl group of **5** with DNA base (adenosine). The NBTI ligand **5** is in stick representation color coded by element, and DNA (cartoon representation) is depicted in orange with adenosine (stick representation) color coded by element.

The secondary linker of **5** forms a salt bridge with the carboxylate of Asp89 (Figure 2). This is a highly conserved residue in DNA gyrases of other bacterial strains^15^ and implies that targeting this key residue can be beneficial in designing broad-spectrum NBTIs. Another feature that emerges from the present crystal structure is that the hydroxyl group in the linker in **5** can form a hydrogen bond with an adenine as a DNA base (distance: 2.8 Å, angles: 126.5° and 115.2°, Figure 4). This particular hydrogen bonding has been seen in previously published crystal structures of NBTIs in complex with DNA gyrase and DNA where a hydroxyl group was present in the NBTI’s linker part (PDB IDs: 4BUL, 4PLB, and 5BS3, Figure S2).^31−33^ Since, this crystal structure is not the only case where such a hydrogen bond exists, we hypothesize that the incorporation of a hydroxyl group into the linker region of NBTIs should systematically be explored in order to position the LHS and increase the target affinity. Namely, with or without an −OH group in the linker region, water molecules are displaced during ternary complex formation, as there is very little room left for water molecules to remain bound. In the unbound state, both adenine amine and ligand −OH are solvated so that the binding event should result in the displacement of solvating water molecules, leading to an enthalpy increase (unfavorable) and entropy increase (favorable). An additional hydrogen bond between the inhibitor and DNA would favor binding by decreasing enthalpy.

We have introduced novel derivatives of mycobacterial DNA gyrase inhibitors with excellent *in vitro* enzyme inhibition profiles and potent antibacterial activity. Our research has yielded the first crystal structure of an *M. tuberculosis* DNA gyrase/DNA/NBTI complex, revealing the binding site of an NBTI as a springboard for future structure-based drug design campaigns and underscoring the potential role of halogen bonds in designing potent NBTIs. Given the urgent demand for new antituberculosis agents, we are confident that the DNA gyrase inhibitors detailed in this study hold significant promise for further refinement. This could lead to the development of innovative drugs aimed at addressing the growing menace of antimycobacterial resistance.

## Supporting Information Available

Residual activity; IC_50_ determination data; protein expression, purification and crystallization procedures; and X-ray data collection and refinement statistics (PDF)

## Accession Codes

The crystal structure is deposited in the Protein Data Bank with the PDB ID: 9FOY.

## Abbreviations

NBTI: novel bacterial topoisomerase inhibitors
topoIV: topoisomerase IV
*M. tuberculosis*: *Mycobacterium tuberculosis*
LHS: left-hand-side
RHS: right-hand-side
