# Supplementary material for: Structural Aspects of Mycobacterium tuberculosis DNA Gyrase Targeted by Novel Bacterial Topoisomerase Inhibitors
Source: ACS Med Chem Lett. 2024 Nov 22;15(12):2164–70. doi: 10.1021/acsmedchemlett.4c00447 (PMC11647683; doi:10.1021/acsmedchemlett.4c00447)
Supplement: Supplementary file 1 — ml4c00447_si_001.pdf [file ml4c00447_si_001.pdf]

# Supplementary information

Structural aspects of *Mycobacterium tuberculosis* DNA gyrase targeted by novel bacterial topoisomerase inhibitors

Maja Kokot [a, b], Martina Hrast Rambaher [b], Lipeng Feng [c,d], Lesley A Mitchenall [c,d], David M Lawson [c,e], Anthony Maxwell [c,d], Tanya Parish [f], Nikola Minovski *[a], Marko Anderluh *[b]

* Corresponding Authors: Nikola Minovski (nikola.minovski@ki.si) and Marko Anderluh (marko.anderluh@ffa.uni-lj.si).

[a] Theory Department, Laboratory for Cheminformatics, National Institute of Chemistry, Hajdrihova 19, 1001 Ljubljana, Slovenia.

[b] Department of Pharmaceutical Chemistry, Faculty of Pharmacy, University of Ljubljana, Aškerčeva cesta 7, 1000 Ljubljana, Slovenia.

[c] Department of Biological Chemistry, John Innes Centre, Norwich Research Park, Norwich NR4 7UH, UK.

[d] Department of Molecular Microbiology, John Innes Centre, Norwich Research Park, Norwich NR4 7UH, UK.

[e] Department of Biochemistry & Metabolism, John Innes Centre, Norwich Research Park, Norwich NR4 7UH, UK.

[f] School of Medicine, University of Washington, Seattle, Washington, USA and Center for Global Infectious Disease Research, Seattle Children's Research Institute, Seattle, Washington, USA.

## Table of content

Percent of *M. tuberculosis* DNA gyrase inhibition of NBTIs at 100 nM concentrations........................3

Figures of the binding site ..........................................................................................................5

Experimental section...................................................................................................................6

References..................................................................................................................................16

**Percent of *M. tuberculosis* DNA gyrase inhibition of NBTIs at 100 nM concentrations.**

**Table S1.** Percent of M. tuberculosis DNA gyrase inhibition of NBTIs at 100 nM concentrations.

| Cmpd | Structure | % Inhibition at 100 nM | Cmpd | Structure | % Inhibition at 100 nM |
|---|---|---|---|---|---|
| **1** | 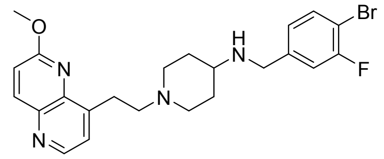 | 84.12 ± 3.60 | **19** | 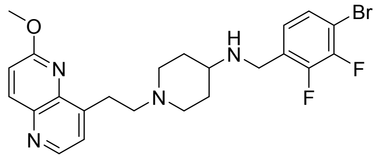 | 29.92 ± 6.02 |
| **2** | 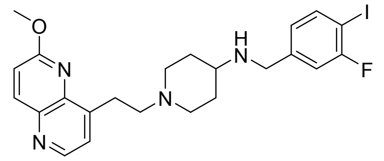 | 80.41 ± 7.17 | **20** | 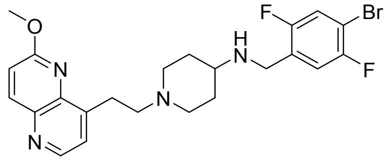 | 43.13 ± 2.75 |
| **3** | 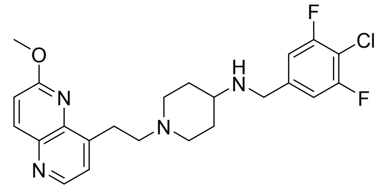 | 90.20 ± 0.45 | **21** | 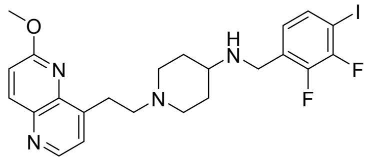 | 14.42 ± 7.17 |
| **4** | 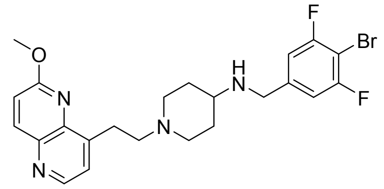 | 99.79 ± 0.22 | **22** | 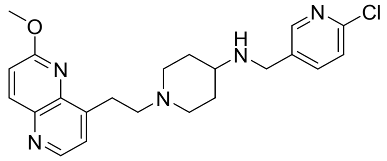 | 27.26 ± 2.41 |
| **5** | 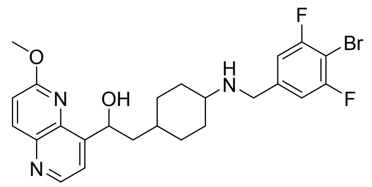 | 95.03 ± 2.46 | **23** | 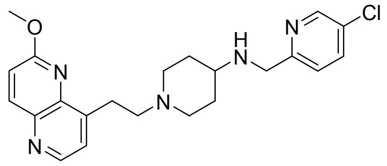 | 0 |
| **6** | 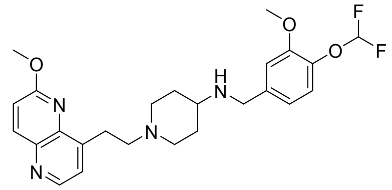 | 0 | **24** | 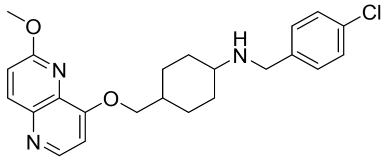 | 4.7 ± 4.7 |
| **7** | 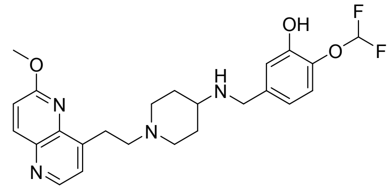 | 3.47 ± 1.66 | **25** | 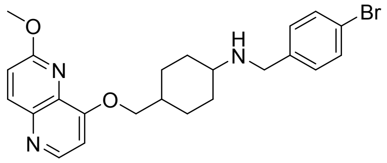 | 0 |
| **8** | 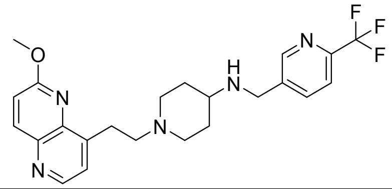 | 27.39 ± 7.04 | **26** | 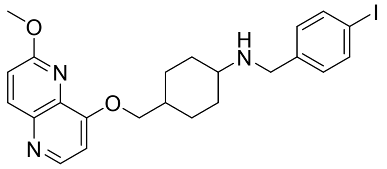 | 9.68 ± 6.59 |

| | | | | | |
|---|---|---|---|---|---|
| **9** | 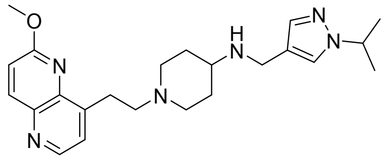 | 7.45 ± 7.45 | **27** | 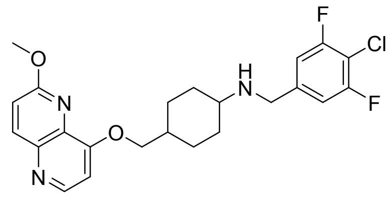 | 41.58 ± 2.84 |
| **10** | 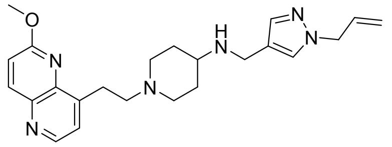 | 5.13 ± 5.13 | **28** | 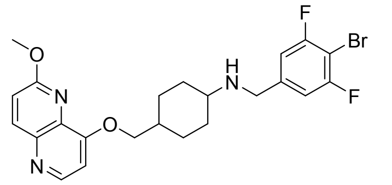 | 23.75 ± 8.50 |
| **11** | 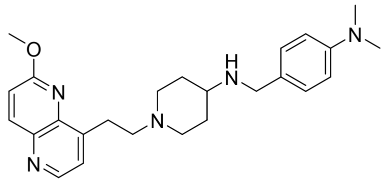 | 0 | **29** | 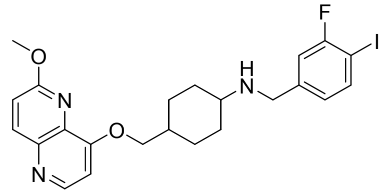 | 27.83 ± 1.23 |
| **12** | 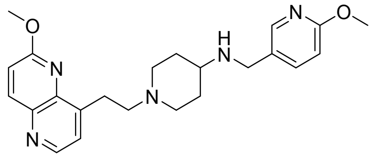 | 0 | **30** | 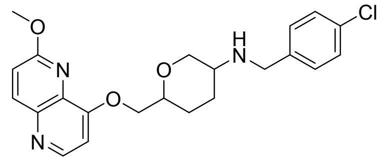 | 0 |
| **13** | 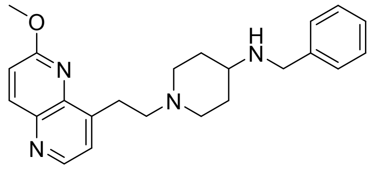 | 0 | **31** | 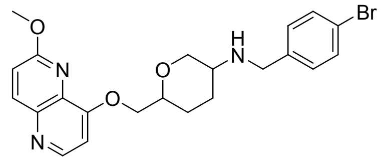 | 0 |
| **14** | 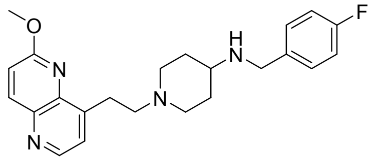 | 0 | **32** | 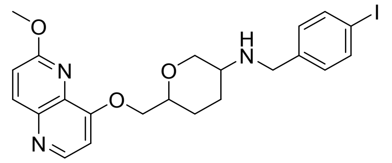 | 0.60 ± 0.60 |
| **15** | 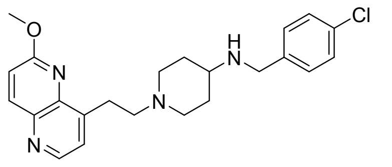 | 44.80 ± 9.60 | **33** | 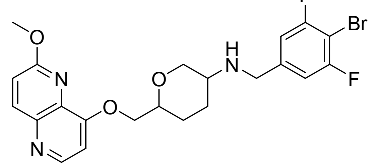 | 19.30 ±1.65 |
| **16** | 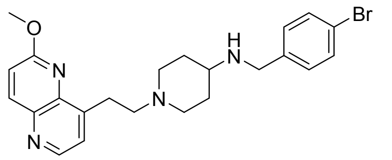 | 64.08 ± 2.29 | **34** | 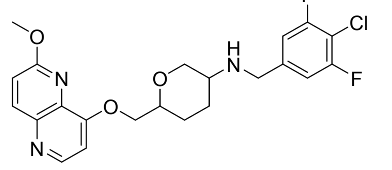 | 4.30 ± 2.84 |
| **17** | 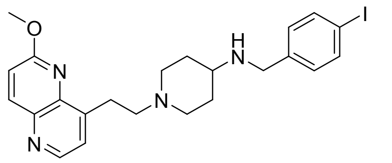 | 63.72 ± 3.40 | **35** | 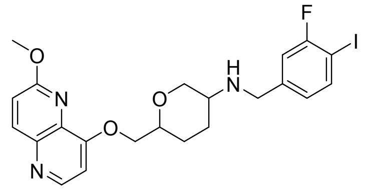 | 20.31 ± 2.81 |
| **18** | 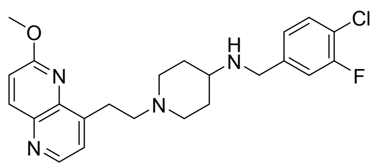 | 52.16 ± 2.65 | **36** | 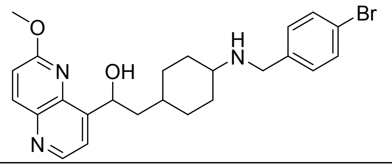 | 24.86 ± 3.89 |

## Figures of the binding site

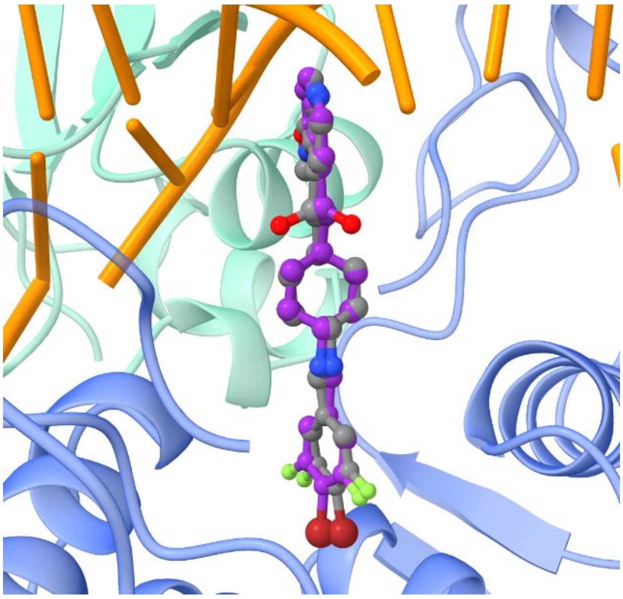

**Figure S1.** The compound is present in the crystal structure as a racemate. One stereoisomer is colored in grey and one is colored in violet.

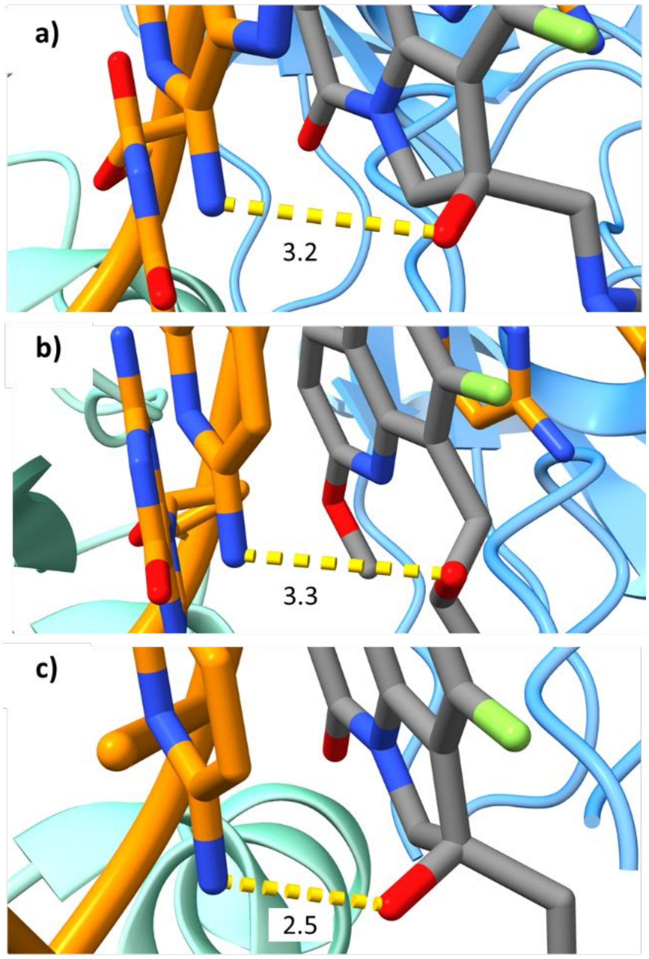


**Figure S2.** Hydrogen bonds in previously published crystal structures of NBTIs in complex with DNA gyrase/DNA where a hydroxyl group was present in the NBTI linker region. **a)** PDB ID: 4BUL;[1] **b)** PDB ID: 4PLB;[2] **c)** PDB ID: 5BS3.[3] Ligands are in sticks representation color coded by element, DNA is orange, adenosine is color coded by element.

## Experimental section

*In vitro DNA gyrase inhibitory activity*

***Determination of $IC_{50}$ values of compounds 1-5 using M. tuberculosis gyrase supercoiling assays***

**Assay**

In all experiments, the activity of the enzyme was determined prior to the testing of the compounds and 1 U defined as the amount of enzyme required to fully supercoil the substrate. This amount of enzyme was used in all subsequent assays. The final DMSO concentration in all the reactions was 1% (v/v). Compounds were serially diluted in DMSO and added to the reaction before the addition of the enzyme. Control compound for all assays was ciprofloxacin.

***M. tuberculosis* gyrase supercoiling assay**

1 U of DNA gyrase was incubated with 0.5 µg of relaxed pBR322 DNA in a 30 µl reaction at 37 °C for 30 min under the following conditions: 40 mM HEPES·KOH (pH 7.6), 10 mM magnesium acetate, 10 mM DTT, 2 mM ATP, 500 mM potassium glutamate and 0.05 mg/ml BSA. Each reaction was stopped by the addition of 30 µl chloroform/iso-amyl alcohol (24:1) and 20 µl Stop Dye (40% sucrose, 100 mM Tris.HCl (pH 7.5), 10 mM EDTA, 0.5 µg/ml bromophenol blue), before being loaded on a 1.0% TAE (Tris.acetate 0.04 mM, EDTA 0.002 mM). Gels run at 70V for 3 hours.

**Data acquisition and analysis**

Bands were visualized by ethidium staining for 10 min, de-stained for 10 min in water and bands were analyzed by gel documentation equipment (Syngene, Cambridge, UK) and quantitated using Syngene Gene Tools software. Raw gel data (fluorescent band volumes) collected from Syngene, GeneTools gel analysis software were converted to a % of the 100% control (the fully supercoiled or relaxed DNA band) and plotted against the inhibitor concentration using SigmaPlot Version 13 (2015). The global curve fit non-linear regression tool was used to calculate $IC_{50}$ data using the following equation: Equation: Exponential Decay, Single, 2 Parameter $f=ae-bx$

**Table S2.** $IC_{50}$ results of *M. tuberculosis* DNA gyrase supercoiling.

| Compound | *M. tuberculosis* gyrase supercoiling $IC_{50}$ (µM) | | |
|---|---|---|---|
| | Assay 1 | Assay 2 | **Average** |
| **1** | 0.397 | 0.330 | **0.363** |
| **2** | 0.155 | 0.056 | **0.105** |
| **3** | 0.093 | 0.091 | **0.092** |
| **4** | 0.089 | 0.069 | **0.079** |
| **5** | 0.097 | 0.096 | **0.096** |
| Gepotidacin | 4.09 | 3.79 | **3.94** |

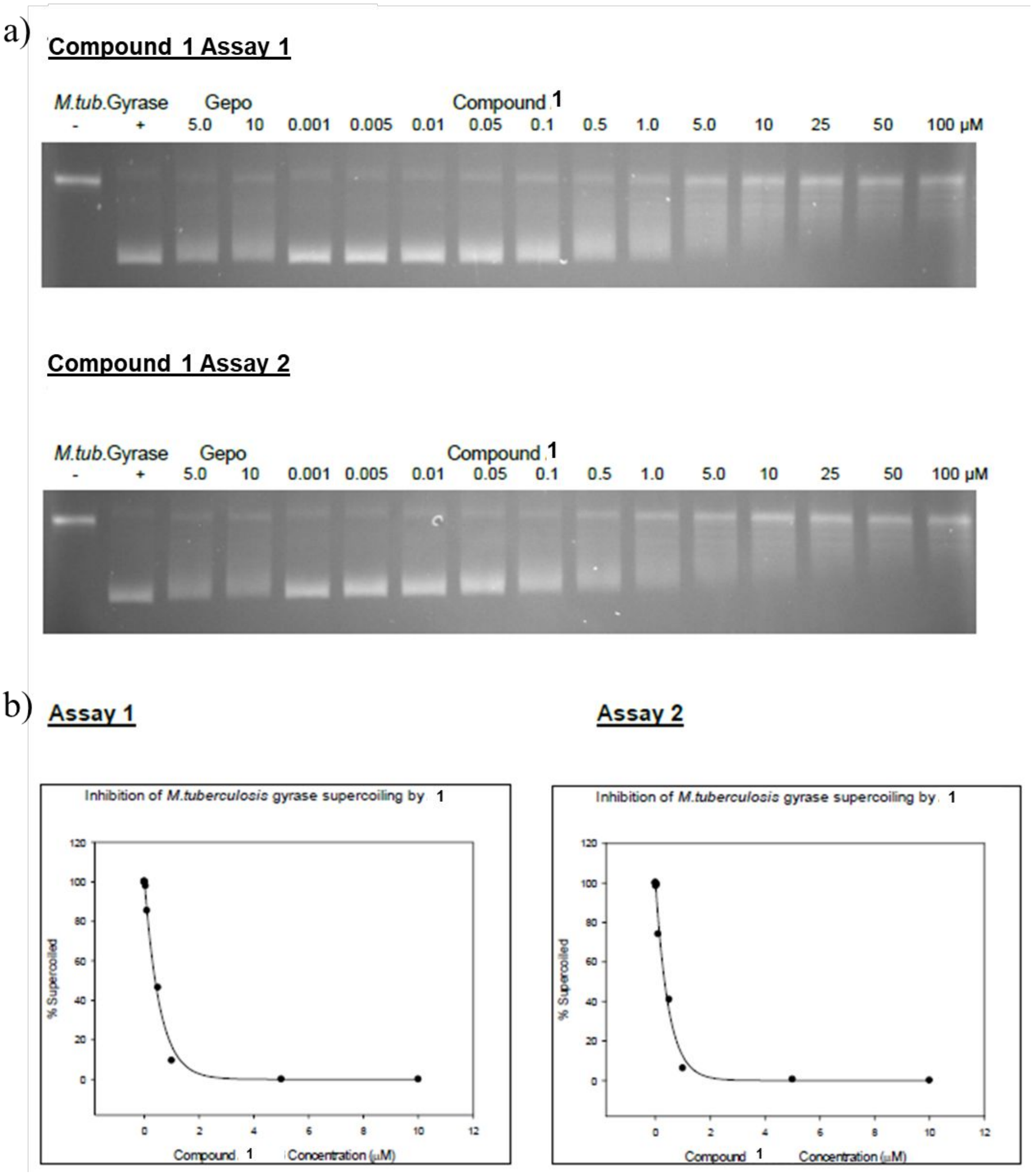


**Figure S3. a)** Gel Image. Inhibition of *M. tuberculosis* DNA gyrase supercoiling by Compound **1**. **b)** DNA gyrase supercoiling inhibition plot with Compound **1**. $IC_{50}$ calculated: Assay 1 = 0.397 μM and Assay 2 = 0.330 μM.

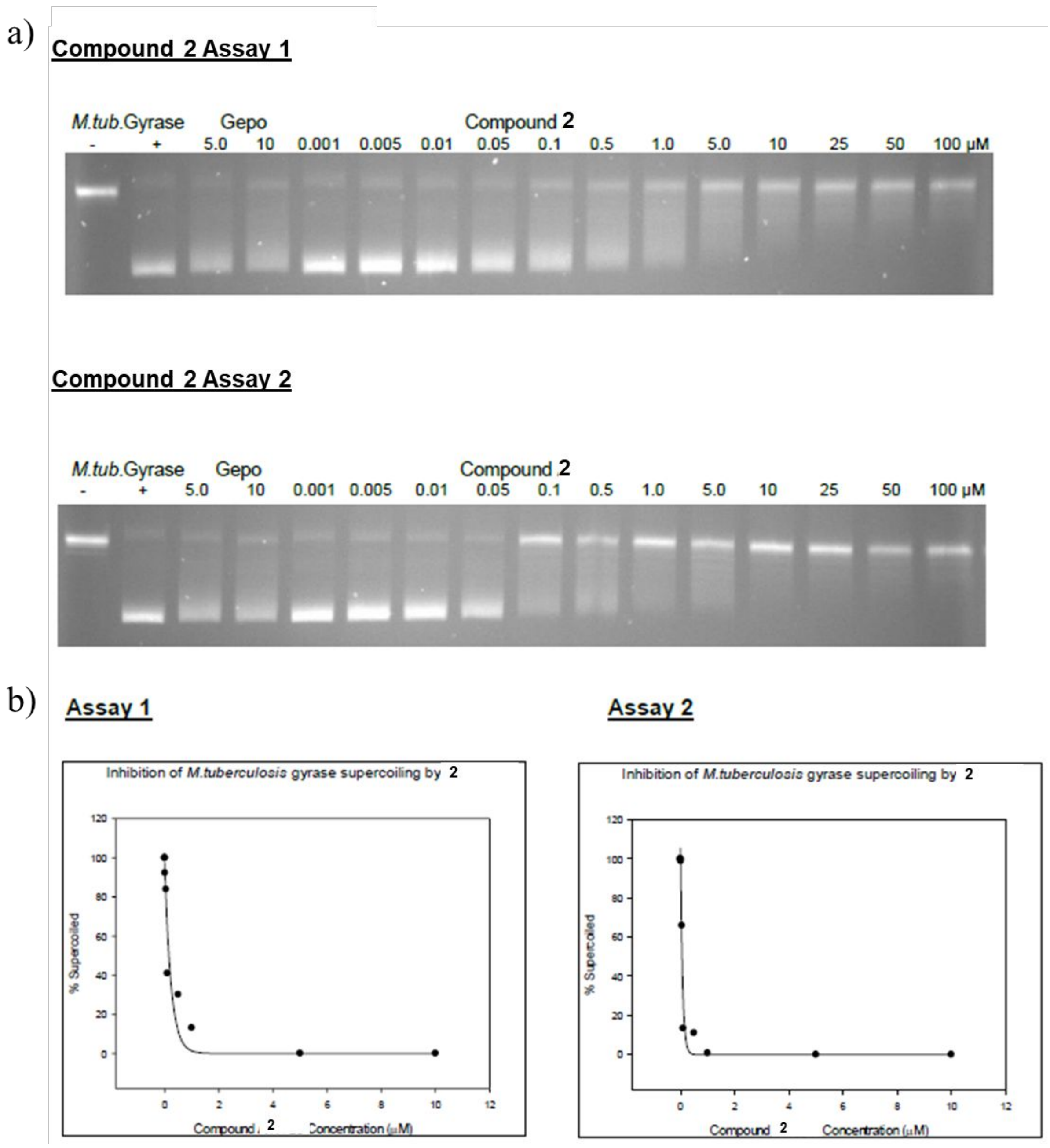


**Figure S4. a)** Gel Image. Inhibition of *M. tuberculosis* DNA gyrase supercoiling by Compound **2**. **b)** DNA gyrase supercoiling inhibition plot with Compound **2**. $IC_{50}$ calculated: Assay 1 = 0.155 µM and Assay 2 = 0.056 µM.

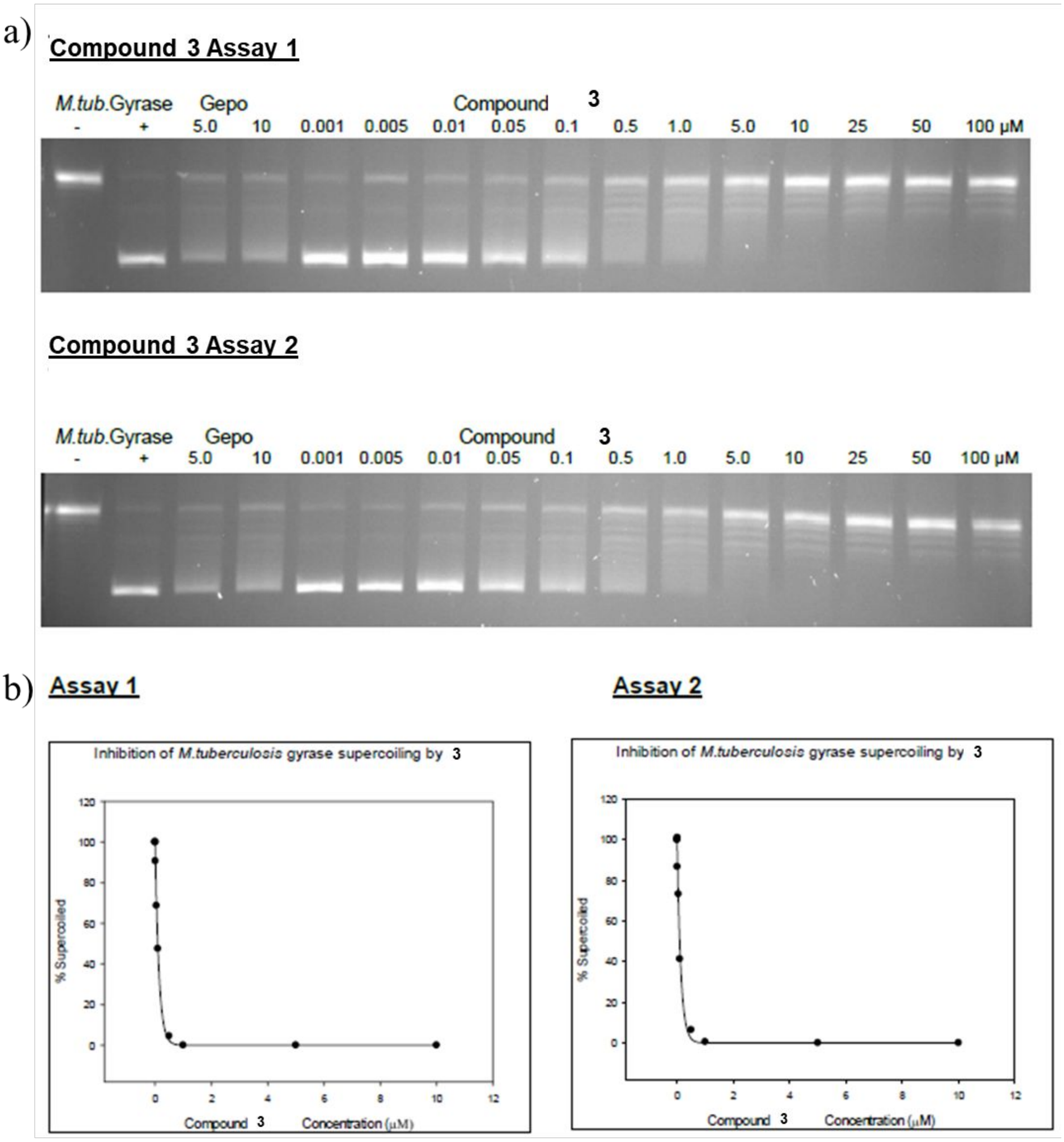


**Figure S5. a)** Gel Image. Inhibition of *M. tuberculosis* DNA gyrase supercoiling by Compound **3**. **b)** DNA gyrase supercoiling inhibition plot with Compound **3**. $IC_{50}$ calculated: Assay 1 = 0.093 µM and Assay 2 = 0.091 µM.

a) b)

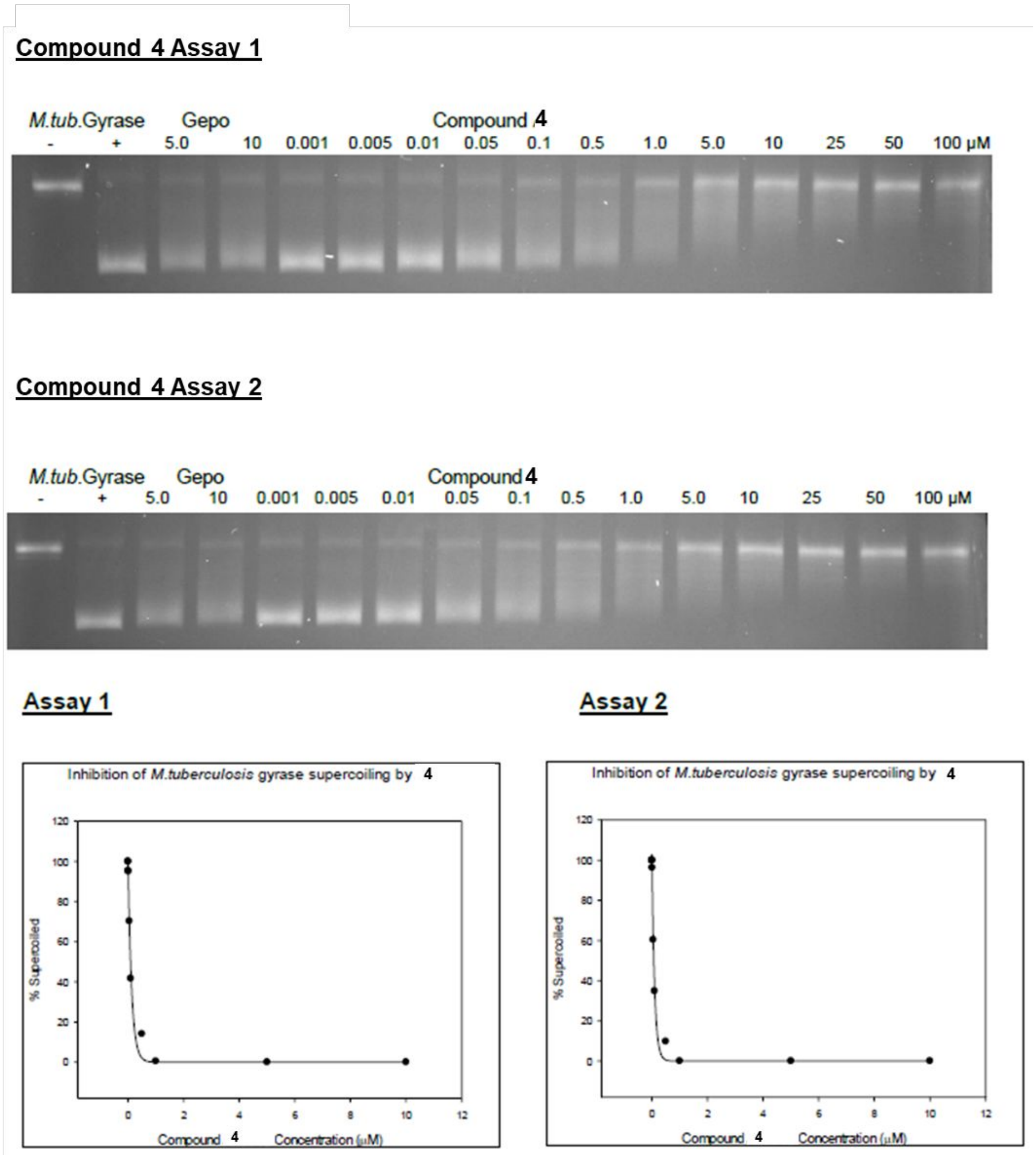


**Figure S6. a)** Gel Image. Inhibition of *M. tuberculosis* DNA gyrase supercoiling by Compound **4**. **b)** DNA gyrase supercoiling inhibition plot with Compound **4**. $IC_{50}$ calculated: Assay 1 = 0.089 μM and Assay 2 = 0.069 μM.

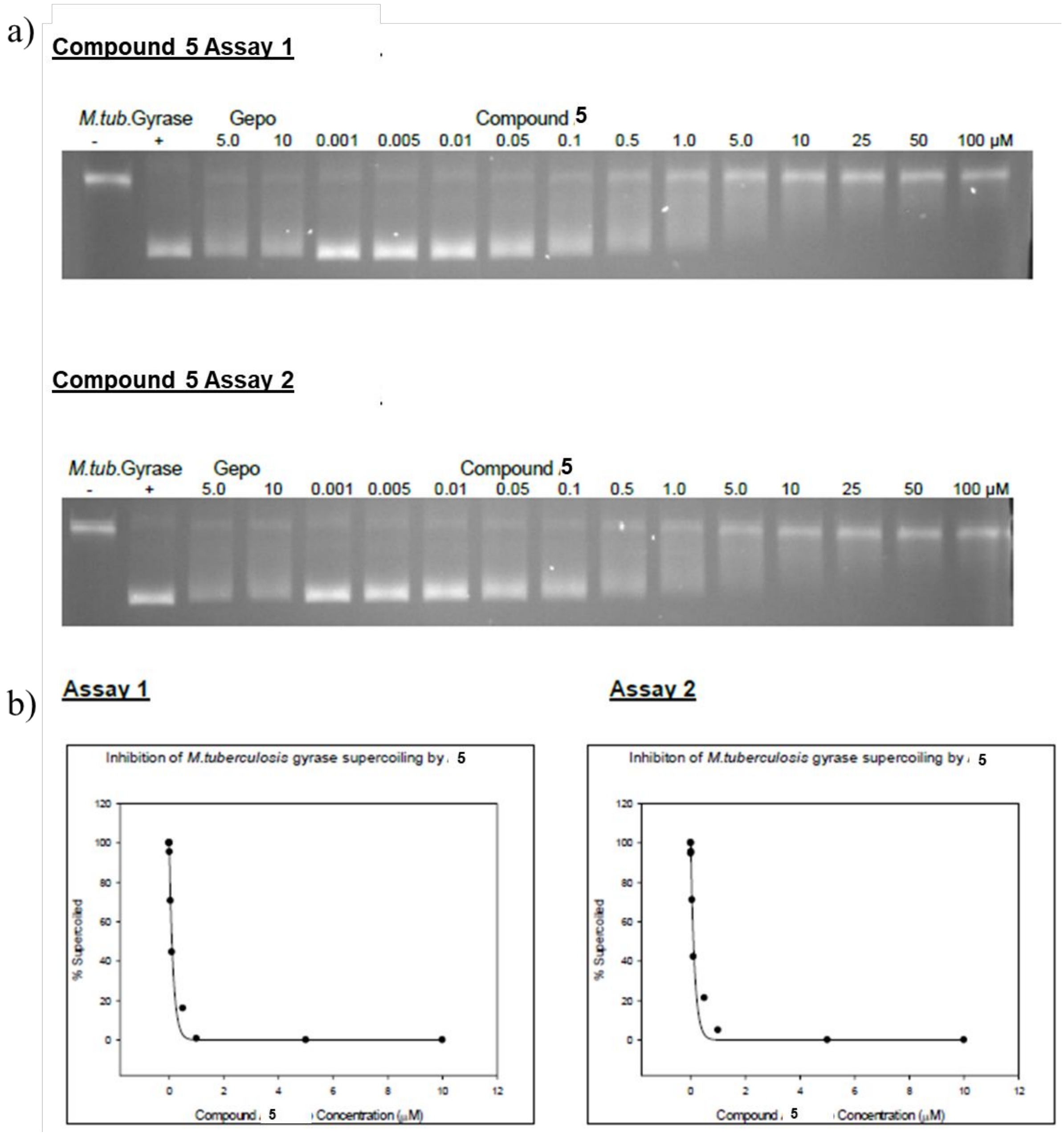


**Figure S7. a)** Gel Image. Inhibition of *M. tuberculosis* DNA gyrase supercoiling by Compound **5**. **b)** DNA gyrase supercoiling inhibition plot with Compound **5**. $IC_{50}$ calculated: Assay 1 = 0.097 μM and Assay 2 = 0.096 μM.

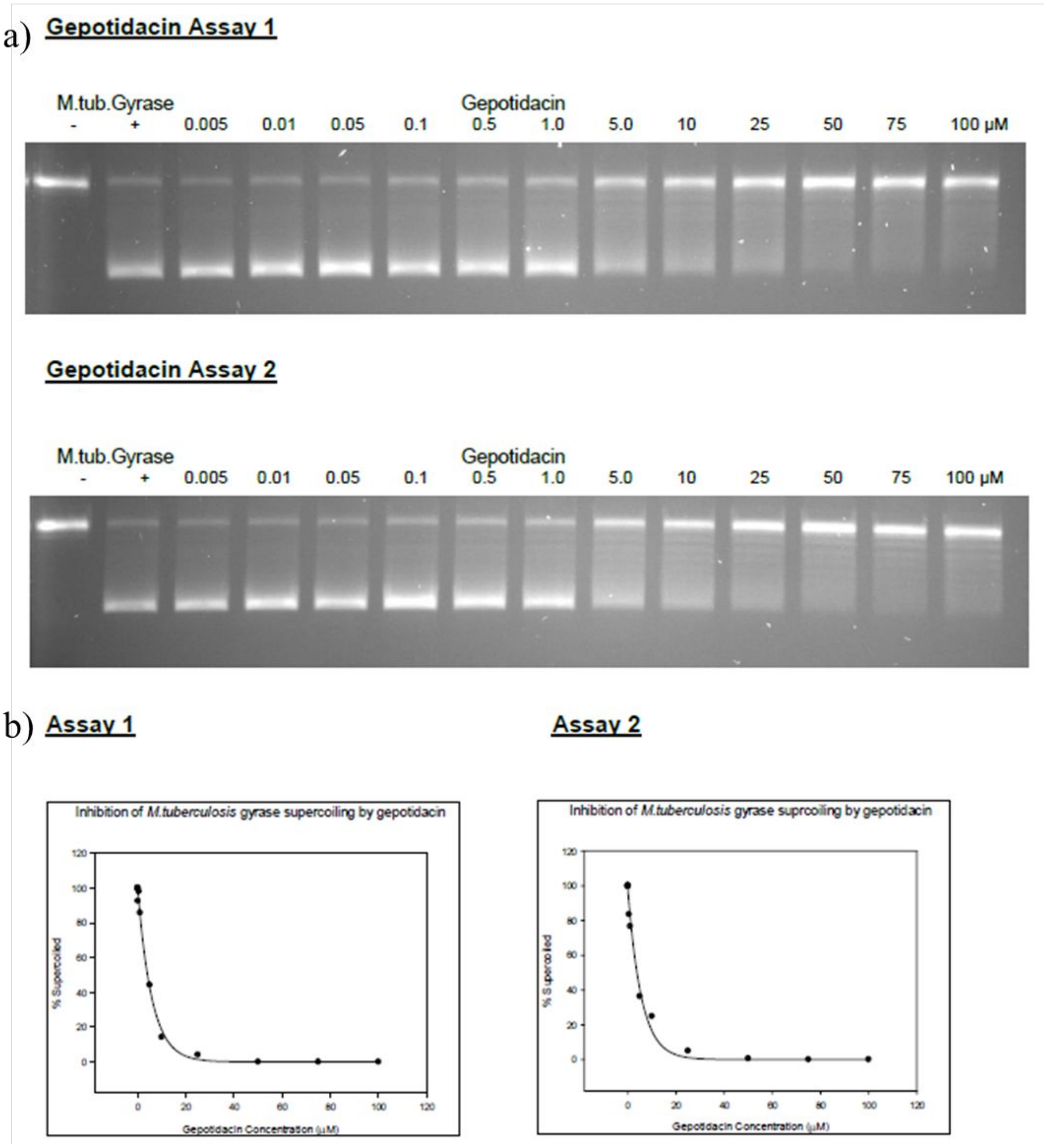


**Figure S8. a)** Gel Image. Inhibition of *M. tuberculosis* DNA gyrase supercoiling by Gepotidacin. **b)** DNA gyrase supercoiling inhibition plot with Gepotidacin. $IC_{50}$ calculated: Assay 1 = 4.09 µM and Assay 2 = 3.79 µM.

*Antituberculosis activity*

*M. tuberculosis* strains H37Rv LP (ATCC25618) and a fluoroquinolone-resistant isolate (LP-FQ-RM9) were cultured in Middlebrook 7H9 supplemented with 10% v/v oleic acid, albumin, dextrose, and catalase (OADC) (Becton Dickinson) and 0.05% w/v Tween 80. Compound activity was determined as described.[4] Briefly, log-phase cultures were diluted to an OD of 0.02, exposed to compounds for 5 days and growth measured by $OD_{590}$. Growth was calculated with reference to controls (DMSO only) and curves fitted using the Levenberg–Marquardt fit. $IC_{50}$ and $IC_{90}$ were calculated as the concentration required to reach 50% and 90% inhibition of growth respectively.

*Protein expression and purification*

The expression construct for the *M. tuberculosis* gyrase core fusion was kindly provided by Tim Blower, and its production is described elsewhere.[5]

After cleavage of the affinity tags, the resultant protein comprised residues 426-675 of GyrB (UniProtKB entry P9WG45) fused directly onto residues 1-500 of GyrA (UniProtKB entry P9WG47), with additional short non-native sequences of SNA and IGSG appended to the N- and C-termini of the polypeptide chain, respectively. Following the final purification step, the protein was concentrated to 36 mg/ml in buffer (20 mM Tris·HCl, pH 7.9 and 100 mM NaCl). DNA oligos (AGCCGTAG and pGTACCTACGGCT) were ordered from Sigma-Aldrich. Each oligo was dissolved in sterile MilliQ water to 8 mM concentration. Equal volumes of each oligo were mixed and annealed in a PCR instrument by heating up the solutions to a starting temperature of 95°C and gradually decreasing the temperature by 12°C every minute.

*Protein crystallization*

Prior to crystallization 10.3 µl of the purified protein at ~36 mg/ml was defrosted and mixed with 20.15 µl of buffer (20 mM Tris·HCl pH 8 and 100 mM NaCl), 1.05 µl of 10 mM compound **5** stock in 100% (v/v) DMSO and 3.5 µl of the 2 mM DNA duplex. This gave 35 µl of the protein:DNA:inhibitor complex. This complex was incubated for 30 mins at 25°C and then centrifuged at 13,000 g in a benchtop centrifuge for 10 minutes prior to setting up the crystallization. All crystallizations were set up using microbatch plates. Crystals were grown using the sitting drop vapor diffusion method from the sample above. Commercially available (Molecular Dimensions, Qiagen) and in-house crystallization screens were set up in MRC2 96-well crystallization plates (Swissci) with drops comprised of 0.3 µL precipitant and 0.3 µL of protein solution using an Oryx 8 liquid handling robot (Douglas Instruments) and then equilibrated against 50 µL of reservoir solution at a constant temperature of 20˚C. Suitable crystals were harvested and mounted in Litholoops (Molecular Dimensions). After transfer to a cryoprotectant comprised of 0.2 M lithium chloride, 0.1 Tris·HCl pH 8.0, 20% (w/v) PEG8000 and 25% (v/v) ethylene glycol, they were flash-cooled by plunging into liquid nitrogen prior to transport to the synchrotron.

*X-ray data collection, processing and structure solution*

X-ray data were recorded on beamline I04 at the Diamond Light Source (Oxfordshire, UK) using an Eiger2 16M detector (Dectris) with the crystal maintained at 100 K by a Cryojet cryocooler (Oxford Instruments). A total of 3600 x $0.1^0$ images were recorded to a maximum resolution of 2.8 Å. The data were integrated and scaled using DIALS[6] and then merged using AIMLESS.[7] The space group was $P2_1$ with cell parameters of $a$ = 82.0, $b$ = 131.7, $c$ = 96.3 Å, $\beta$ = 97.2°. Data collection statistics are summarized in Table S3.

The majority of the downstream analysis was performed through the CCP4i2 graphical user interface.[8] The structure was solved automatically by molecular replacement using PHASER[9] and refined using REFMAC5,[10] as implemented through the DIMPLE pipeline,[11] with only the protein components of a previously solved *M. tuberculosis* DNA gyrase core fusion complex (PDB accession 5BTA) as the template coordinates. This gave one copy of the biologically relevant fusion homodimer in the asymmetric unit (ASU), with initial $R_{work}$ and $R_{free}$ values of 0.337 and 0.372, respectively. At this stage, inspection in COOT[12] revealed very clear positive difference electron density for the missing DNA components, and a very strong density peak lying on the non-crystallographic two-fold symmetry axis corresponding to the position of the ligand halide atom in the related *S. aureus* DNA gyrase ternary complex with DNA and inhibitor AMK12 (PDB accession 6Z1A). The DNA was added manually in COOT, including phosphotyrosine links between the 5' ends of the longer DNA fragments and the two copies of Tyr123, the active site tyrosine. Following further refinement, the density was sufficiently clear to add the ligand too; starting coordinates and a ligand restraint dictionary for **5** were prepared using AceDRG.[13] The ligand has a single chiral center and is synthesized as a mixture of R- and S-stereoisomers. Modelling **5** as a 1:1 racemic mixture gave the best fit to the map with negligible residual density.

The statistics of the final refined model, including validation output from MolProbity,[14] are shown in Table S3. Omit $m\mathrm{F}_{obs}$-$D\mathrm{F}_{calc}$ difference electron density for the bound ligand was calculated using phases from the final model without

the ligand after the application of small random shifts to the atomic coordinates, re-setting temperature factors, and re-refining to convergence. The crystallographic evidence for the ligand is shown with the "omit density" map in the Figure S9.

**Table S3.** X-ray data collection and refinement statistics

| | |
|---|---|
| Inhibitor | AMK32b |
| Data collection | |
| Beamline | I04 Diamond Light Source, UK |
| Wavelength (Å) | 0.9537 |
| Detector | Eiger2 X 16M |
| Resolution range (Å) | 69.23 – 2.80 (2.89 – 2.80) |
| Space Group | $P2_1$ |
| Cell parameters (Å/°) | $a$ = 82.0, $b$ = 113.7, $c$ = 96.3, $\beta$ = 97.2 |
| Total no. of measured intensities | 355596 (31268) |
| Unique reflections | 50035 (4567) |
| Multiplicity | 7.1 (6.8) |
| Mean $I/\sigma(I)$ | 4.9 (0.9) |
| Completeness (%) | 100.0 (100.0) |
| $R_{merge}$ [a] | 0.414 (1.731) |
| $R_{meas}$ [b] | 0.447 (1.875) |
| $CC_{½}$ [c] | 0.959 (0.414) |
| Wilson $B$ value (Å$^2$) | 29.5 |
| Refinement | |
| Resolution range (Å) | 69.33–2.80 (2.87-2.80) |
| Reflections: working/free[d] | 47469/2542 |
| $R_{work}$/ $R_{free}$ [e] | 0.279/0.304 (0.370/0.383) |
| Ramachandran plot: favoured/allowed/disallowed[f] (%) | 96.3/3.3/0.4 |
| R.m.s. bond distance deviation (Å) | 0.004 |
| R.m.s. bond angle deviation (º) | 1.148 |
| No. protein residues | A/B chains:730 |
| No. DNA bases | E/G chains:8; F/H chains:12 |
| No. of AMK32b molecules | 1 |
| Mean $B$ factors: protein/DNA/AMK32b/overall (Å$^2$) | 44/42/38/41 |
| RSCC score[g] for AMK32b | 0.89/90 |
| Accession code | 9FOY |

Values in parentheses are for the outer resolution shell.

[a] $R_{merge} = \sum_{hkl} \sum_i |I_i(hkl) - \langle I(hkl)\rangle| / \sum_{hkl} \sum_i I_i(hkl)$.

[b] $R_{meas} = \sum_{hkl} [N/(N-1)]^{1/2} \times \sum_i |I_i(hkl) - \langle I(hkl)\rangle| / \sum_{hkl} \sum_i I_i(hkl)$, where $I_i(hkl)$ is the $i$th observation of reflection $hkl$, $\langle I(hkl)\rangle$ is the weighted average intensity for all observations $i$ of reflection $hkl$ and $N$ is the number of observations of reflection $hkl$.

[c] $CC_{½}$ is the correlation coefficient between symmetry equivalent intensities from random halves of the dataset.

[d] The data set was split into "working" and "free" sets consisting of 95 and 5% of the data respectively. The free set was not used for refinement.
[e] The R-factors $R_{work}$ and $R_{free}$ are calculated as follows: $R = \sum(| F_{obs} - F_{calc} |)/\sum| F_{obs} |$, where $F_{obs}$ and $F_{calc}$ are the observed and calculated structure factor amplitudes, respectively.
[f] From MolProbity. [14]
[g] From the PDB validation server. The two values correspond to the two enantiomers which were each modelled with 0.5 occupancy.

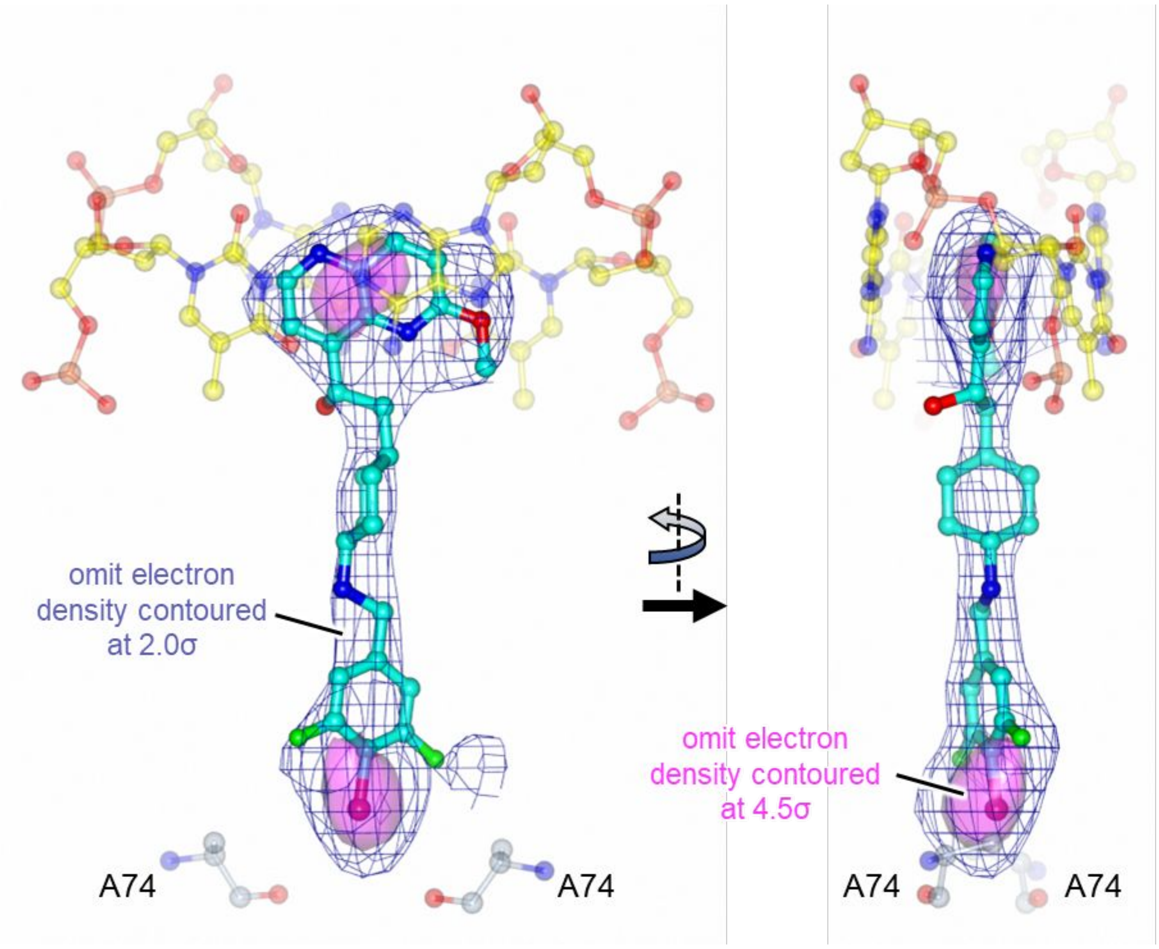


**Figure S9. Crystallographic evidence supporting the placement, orientation and conformation of compound 5 in the complex with DNA gyrase and DNA.** Orthogonal views showing "omit" $m\mathrm{F}_{obs}$-$D\mathrm{F}_{calc}$ difference electron density for the bound ligand (cyan carbons) calculated at 2.8-Å resolution and contoured at 2.0σ (dark blue mesh) and at 4.5σ (magenta semi-transparent surface). Note that the latter highlights the confidence in the placement of the bromine atom. This is shown in the context of the neighboring DNA base pairs (yellow carbons) and the two Ala74 residues (grey carbons). For clarity, only the *S* enantiomer is shown.

## References


(1) Miles, T. J.; Hennessy, A. J.; Bax, B.; Brooks, G.; Brown, B. S.; Brown, P.; Cailleau, N.; Chen, D.; Dabbs, S.; Davies, D. T.; Esken, J. M.; Giordano, I.; Hoover, J. L.; Huang, J.; Jones, G. E.; Sukmar, S. K. K.; Spitzfaden, C.; Markwell, R. E.; Minthorn, E. A.; Rittenhouse, S.; Gwynn, M. N.; Pearson, N. D. Novel Hydroxyl Tricyclics (e.g., GSK966587) as Potent Inhibitors of Bacterial Type IIA Topoisomerases. *Bioorg. Med. Chem. Lett.* **2013**, *23* (19), 5437–5441. https://doi.org/10.1016/j.bmcl.2013.07.013.

(2) Singh, S. B.; Kaelin, D. E.; Wu, J.; Miesel, L.; Tan, C. M.; Meinke, P. T.; Olsen, D.; Lagrutta, A.; Bradley, P.; Lu, J.; Patel, S.; Rickert, K. W.; Smith, R. F.; Soisson, S.; Wei, C.; Fukuda, H.; Kishii, R.; Takei, M.; Fukuda, Y. Oxabicyclooctane-Linked Novel Bacterial Topoisomerase Inhibitors as Broad Spectrum Antibacterial Agents. *ACS Med. Chem. Lett.* **2014**, *5* (5), 609–614.

(3) Singh, S. B.; Kaelin, D. E.; Wu, J.; Miesel, L.; Tan, C. M.; Black, T.; Nargund, R.; Meinke, P. T.; Olsen, D. B.; Lagrutta, A.; Lu, J.; Patel, S.; Rickert, K. W.; Smith, R. F.; Soisson, S.; Sherer, E.; Joyce, L. A.; Wei, C.; Peng, X.; Wang, X.; Fukuda, H.; Kishii, R.; Takei, M.; Takano, H.; Shibasaki, M.; Yajima, M.; Nishimura, A.; Shibata, T.; Fukuda, Y. Tricyclic 1,5-Naphthyridinone Oxabicyclooctane-Linked Novel Bacterial Topoisomerase Inhibitors as Broad-Spectrum Antibacterial Agents-SAR of Left-Hand-Side Moiety (Part-2). *Bioorganic Med. Chem. Lett.* **2015**, *25* (9), 1831–1835.

(4) Ollinger, J.; Bailey, M. A.; Moraski, G. C.; Casey, A.; Florio, S.; Alling, T.; Miller, M. J.; Parish, T. A Dual Read-out Assay to Evaluate the Potency of Compounds Active against Mycobacterium Tuberculosis. *PLoS One* **2013**, *8* (4), e60531. https://doi.org/10.1371/journal.pone.0060531.

(5) Blower, T. R.; Williamson, B. H.; Kerns, R. J.; Berger, J. M. Crystal Structure and Stability of Gyrase-Fluoroquinolone Cleaved Complexes from Mycobacterium Tuberculosis. *Proc. Natl. Acad. Sci. U. S. A.* **2016**, *113* (7), 1706–1713. https://doi.org/10.1073/pnas.1525047113.

(6) Winter, G.; Waterman, D. G.; Parkhurst, J. M.; Brewster, A. S.; Gildea, R. J.; Gerstel, M.; Fuentes-Montero, L.; Vollmar, M.; Michels-Clark, T.; Young, I. D.; Sauter, N. K.; Evans, G. DIALS: Implementation and Evaluation of a New Integration Package. *Acta Crystallogr. Sect. D, Struct. Biol.* **2018**, *74* (Pt 2), 85–97. https://doi.org/10.1107/S2059798317017235.

(7) Evans, P. R.; Murshudov, G. N. How Good Are My Data and What Is the Resolution? *Acta Crystallogr. D. Biol. Crystallogr.* **2013**, *69* (Pt 7), 1204–1214. https://doi.org/10.1107/S0907444913000061.

(8) Potterton, L.; Agirre, J.; Ballard, C.; Cowtan, K.; Dodson, E.; Evans, P. R.; Jenkins, H. T.; Keegan, R.; Krissinel, E.; Stevenson, K.; Lebedev, A.; McNicholas, S. J.; Nicholls, R. A.; Noble, M.; Pannu, N. S.; Roth, C.; Sheldrick, G.; Skubak, P.; Turkenburg, J.; Uski, V.; von Delft, F.; Waterman, D.; Wilson, K.; Winn, M.; Wojdyr, M. CCP4i2: The New Graphical User Interface to the CCP4 Program Suite. *Acta Crystallogr. Sect. D, Struct. Biol.* **2018**, *74* (Pt 2), 68–84. https://doi.org/10.1107/S2059798317016035.

(9) McCoy, A. J.; Grosse-Kunstleve, R. W.; Adams, P. D.; Winn, M. D.; Storoni, L. C.; Read, R. J. Phaser Crystallographic Software. *J. Appl. Crystallogr.* **2007**, *40* (Pt 4), 658–674. https://doi.org/10.1107/S0021889807021206.

(10) Murshudov, G. N.; Skubák, P.; Lebedev, A. A.; Pannu, N. S.; Steiner, R. A.; Nicholls, R. A.; Winn, M. D.; Long, F.; Vagin, A. A. REFMAC5 for the Refinement of Macromolecular Crystal Structures. *Acta Crystallogr. D. Biol. Crystallogr.* **2011**, *67* (Pt 4), 355–367. https://doi.org/10.1107/S0907444911001314.

(11) Wojdyr, M.; Keegan, R. M.; Winter, G.; Ashton, A. W. DIMPLE- a Pipeline for the Rapid Generation of Difference Maps from Protein Crystals with Putatively Bound Ligands. *Acta Crystallogr. Sect. A* **2013**, *69*.

(12) Emsley, P.; Cowtan, K. Coot: Model-Building Tools for Molecular Graphics. *Acta Crystallogr. D. Biol. Crystallogr.* **2004**, *60* (Pt 12 Pt 1), 2126–2132. https://doi.org/10.1107/S0907444904019158.

(13) Long, F.; Nicholls, R. A.; Emsley, P.; Graæulis, S.; Merkys, A.; Vaitkus, A.; Murshudov, G. N. AceDRG: A Stereochemical Description Generator for Ligands. *Acta Crystallogr. Sect. D, Struct. Biol.* **2017**, *73* (Pt 2), 112–122. https://doi.org/10.1107/S2059798317000067.

(14) Davis, I. W.; Leaver-Fay, A.; Chen, V. B.; Block, J. N.; Kapral, G. J.; Wang, X.; Murray, L. W.; Arendall, W. B. 3rd; Snoeyink, J.; Richardson, J. S.; Richardson, D. C. MolProbity: All-Atom Contacts

and Structure Validation for Proteins and Nucleic Acids. *Nucleic Acids Res.* **2007**, *35* (Web Server issue), W375-83. https://doi.org/10.1093/nar/gkm216.
